# Serial follow-up of biventricular function, exercise capacity and NT-proBNP measurements in repaired tetralogy of Fallot: is there a role for MR stress imaging?

**DOI:** 10.1186/1532-429X-13-S1-P194

**Published:** 2011-02-02

**Authors:** Saskia E Luijnenburg, Jochem van den Berg, Adriaan Moelker, Jolien W Roos-Hesselink, Ad JJC Bogers, Yolanda B de Rijke, Barbara JM Mulder, Hubert W Vliegen, Willem A Helbing

**Affiliations:** 1 Erasmus MC - Sophia Children's Hospital, Rotterdam, Netherlands; 2 Erasmus MC, Rotterdam, Netherlands; 3 Academic Medical Center, Amsterdam, Netherlands; 4 Leiden University Medical Center, Leiden, Netherlands

## Aim

To evaluate the natural course of biventricular size and function in patients with repaired tetralogy of Fallot (TOF), in relation to exercise capacity and NT-proBNP, and to study the potential clinical value of magnetic resonance imaging (MRI) during dobutamine stress.

## Background

Longstanding pulmonary regurgitation (PR) in patients with TOF is associated with adverse outcome. Serial follow-up data is limited in TOF patients operated on according to current surgical strategies and may provide insight in the natural course of right ventricular (RV) size and function. This could be useful in decisions on optimal timing of pulmonary valve replacement (PVR).

## Methods

In 36 TOF patients, serial follow-up of MRI at rest and during dobutamine stress, exercise capacity, and NT-proBNP levels was assessed. Analysis was performed based on RV end-diastolic volume (EDV): subgroup I (n=15): RVEDV < 150 ml/m^2^, subgroup II (n=12): RVEDV ≥ 150 ml/m^2^. A third subgroup consisted of 9 patients who had undergone a PVR during follow-up.

## Results

Median time between baseline and follow-up studies was 5.1 yrs (4.1 - 7.4 yrs). In subgroup II, RV volumes and PR increased significantly over time (RVEDV from 163 ± 25 ml/m^2^ to 183 ± 29 ml/m^2^); these parameters did not change in subgroup I. Biventricular function remained stable during follow-up in all subgroups. Biventricular contractile reserve was preserved in all patients. VO_2_ max. decreased during follow-up, particularly in subgroup II, but this was not statistically significant. After PVR, RV volumes and PR decreased significantly; RVEF and LV contractile reserve increased. A lower RV contractile reserve at baseline correlated with a larger RVEDV at follow-up and with an interstudy decrease in VO_2_ max. NT-proBNP levels were significantly higher than in healthy controls (at follow-up 13 ± 10 pmol/l (patients) vs. 4 ± 2 pmol/l (controls)) but did not change over time and were not different between the subgroups.

## Conclusion

RV volumes and PR percentage increased significantly over time, but only in TOF patients who had a RVEDV ≥ 150 ml/m^2^ at baseline. Exercise capacity tended to decrease at 5-year follow-up, but biventricular function, biventricular contractile reserve, and NT-proBNP remained stable, irrespective of baseline MRI measurements. Baseline RV MR stress measurements correlated with important follow-up measurements of RV size and exercise capacity. Stress MRI showed clinically relevant changes after PVR. Stress imaging might be of additional value in the follow-up of TOF patients. In our patients, NT-proBNP provided limited additional information during follow-up.

